# 

**Published:** 1885-12

**Authors:** 


### International Medical Congress.

The ninth annual meeting of this body will be held in Washington City, beginning on the first Monday of September, 1887, and will continue six or eight days.

This meeting occurs in this country upon the invitation of the medical profession, extended at the last meeting of the body in Copenhagen, in 1884, and the invitation was accepted. Some preparation for the meeting was begun about a year ago, but for reasons which need not here be specified, some interruption of this preparatory work was made within the last six months. Such division of sentiment seemed at one time, to prevail that the success of the congress appeared to be in jeopardy; that embarrassment seems in a measure to have been removed, and the work of preparation seems to now go forward in a manner that promises a successful congress. Early in the course of the initiative work a section on oral and dental surgery was established, that, with two or three other sections was for a time dropped from the list, but within two months afterward the dental section was restored, with its organization, so far as it had been previously effected. A meeting of the executive committee of the congress, which consists of the president, the secretary-general, the treasurer, the chairman the finance committee, and the presidents of the various sections, was held in New York, November 18th, for further perfecting the organization of the congress. The work accomplished at this meeting was very encouraging indeed; the utmost harmony prevailed and many letters of encouragement and co-operation were read. Many particulars of the work of the congress were considered and arranged for. The dental section ought to be made eminently successful, if the dentists of the country give the co-operation and support, which they ought to do; there is certainly no occasion for opposition. If there are any who are not in sympathy with the section and its work, there is no reason why they should make opposition. The work of this section will not in any way antagonize or interfere with any other work in the profession. On every account it should have the hearty co-operation and support of every dentist in the country.

The following from Professor N. S. Davis, Editor of the Journal of the American Medical Association, and Secretary-General of the International Medical Congress, is pertinent, and should set at rest the misconceptions and doubts that seem to be entertained by some, in regard to the Section on Oral and Dental Surgery. He says:

So far as we have observed, the journals in different parts of the country, devoted to the interests of Oral and Dental Science and practice, give expressions of cordial approval of the present preliminary organization of the Congress, and especially of the organization of the Section of Oral and Dental Surgery. The only exception we have noticed is that of the *Independent Practitioner*, published at Buffalo, whose editor, in the December number, has the following singular statement: "Following this, (the meeting of the committee on organization in Chicago, in June last), came the announcement that the Section of Dental and Oral Surgery was, by the reorganized committee, abolished, and that dentists would not be welcomed to the Congress upon the ground that such medical men as had devoted their lives to the consideration of *diseases* of the Oral cavity were not engaged in the legitimate practice of any branch of the healing art." It is a sufficient answer to all this, to simply state the fact that no such announcement was ever authorized by the "reorganized committee," nor by any other party representing the American Medical Association. On the contrary the latter body had fully recognized the relations of Dental and Oral Surgery to the general field of medicine and surgery, several years since by organizing and maintaining a section in that department on the same level with all its other sections. The simple facts are, that the Committee of Arrangements at its first meeting, in June, found nineteen sections organized, which it was then thought was a greater number than could be well accommodated with convenient rooms in Washington, and more than had been provided for at any of the preceding Congresses. Solely for the purpose of reducing the number, propositions were made and temporarily adopted to discontinue the Sections of Mental and Nervous diseases, of Gynecology, and of Dental and Oral Surgery, leaving the workers in those departments to take their places in the Sections of General Medicine, Obstetrics and General Surgery respectively. We say temporarily adopted, because neither the revision of the rules nor of the Sections was completed and finally adopted by the committee until its second meeting in September, when, after more full consideration, all the three Sections, the discontinuance of which, had been proposed were retained without a dissenting vote in the committee; that of Dental and Oral Surgery, without the change of a single officer. And there is every indication that the Section will be sustained with a degree of enthusiasm that will not only do credit to the many scientific workers in that department, but will also aid much in restoring that department to its proper place, as a legitmate branch of the healing art, and much to the benefit of both specialists and general practitioners. And it is to be hoped that this Section will be made so eminently successful that no Congress hereafter will be considered complete without it, for we agree fully with the renowned Virchow, that no one department can be isolated altogether without detriment to the whole.

### INDEX TO VOLUME XXXIX.

Address—President's, before Northwestern Dental Asso.. 8
" Pres. Miss. Val. Asso... 230
" of Welcome—Southern Dental Association............ 266
" before Kentucky State Dental Association............ 383
" Pres. of British Dental Association ...................... 568
Absorption of Roots of Temporary Teeth ..................... 64
Anæsthetics, Precautions in Administration of.................. 85
Antiseptics.........................92, 141
Alumni ................................ 107
Aseptol, the New Antiseptic...... 141
Association of Dental Examiners, National...... ............. 157
" Southern Dental..... 158
Antrum of Highmore, Tooth in 202
Association, Miss. Valley......... 213
Artificial Crown.................... 297
Association, American Dental 368, 373, 477, 488.............. 533
American Medical Association, Proceedings..................... 386
Anæsthetics ........................429, 577
Abscesses, Treatment of Deep-seated ............................ 453
Anæsthesia............................ 458
Asso. Dental College Faculties.. 481
Anatomy, Histology and Microscopy ............................ 515
Alveolar Dental Membrane...... 523
Abscess, Pathology of............. 585
" and Dead Teeth......... 590
Agriculturist, American........... 532
Association, British Dent......... 568
Bromine .............................. 199
Bleaching Teeth..................... 250
Boston Dental College............ 420
Bridgework .......................... 510
Bibliography........530, 532, 576, 584
Cervical Border, Protection of the ................ ............. 5
Correspondence......37, 149, 203, 577
Cocaine........46, 57, 97, 138, 144, 147, 148, 185, 188, 259, 387, 448, 577, 513.................... 580
Camphor Inhalations in Coryza.. 44
Calculus, Salivary................. 59
Catarrh, Chronic Nasal........... 107
Canabis Indica..................187, 347
Carbuncles............................ 197
Chlorine .............................. 199
Crowns, Artificial.................... 297
Cancer of the Tongue............. 331
Case, Unusual........................ 372
" a Peculiar..................... 374
Crown Work.......................... 382
College, Baltimore Dental........ 415
" Ohio Dental.............. 415
" Pennsylvania............. 416
" Dental, of University of Tennessee ........................ 416
" Philadelphia.............. 417
" Dental, University of Michigan........................ 417
" Pennsylvania, Department of Dentistry............ 418
" New York, of Dentistry 419
" University of Maryland Dent. Department............ 419
" Boston ..................... 420
" Indiana .................... 420
" Chicago.................... 421
" Minnesota, Hospital, Dent. Department............ 421
" Missouri ................... 421
" Kansas City............... 421
" California, University of Dent. Department......... 422
" National University, Dent. Department............ 422
Children in Dental Chair, Management of........................ 443
College Faculties, Association of Dent.......................... 481
Caries, Etiology of.................. 515
Dentistry, Operative......10, 238, 509

Dentine over Endangered Pulps, On Assuring Healthy......... 75
Dental Institute in Berlin, New. 92
Diplomas, Sale of American in Europe .......................... 132
Dental Diseases, Collective Investigation of..................... 136
" Examiners' National Association of...........157, 364, 373
" Association Southern..... 158
" Society, Ohio.....159, 480, 528, 529 ........................ 582
" Prothesis...................... 596
Diet and the Teeth.................. 176
Development Hypothesis......... 180
Disinfectants ......................199, 473
Dental Law...208, 403, 405, 408, 411
Dyspepsia ............................. 252
Dentistry, Application of, to Detection of Crime............ 254
Dental Literature, Report on..... 315
Dentistry, Early..................... 331
Dental Economics...............342, 345
Dental Education, Popular...... 347
Dental Association, American... 368, 373, 477 ..................... 488
Deformity, Oral..................... 377
Delaware Dental Law.............. 403
Damage, Artificial Teeth may do 475
Dental Membrane, Alveolar...... 523
Dentistry, Principles and Practice of.............................. 530
Dentistry, Past, Present, Future. 563
Dentition, Diarrhea During..... 581
Dentists' Leg......................... 581
Electrical Surgery ................. 47
Editorial.........53, 104, 155, 208, 260, 334, 368, 425, 477, 529, 582
Education Dental, Progress of.. 162, 166 .......................... 168
Education Dental, Popular...... 347
Eugenol...........................185, 227
Extraction of Teeth................ 255
Economics, Dental............342, 345
Extracting Free...................... 352
Educational ......................373, 415
Earthy Phosphates.................. 491
Enamel, Pathology of............. 515
Etiology of Caries.................. 515
Furnace, Gas......................79, 119
Face, Human......................... 88
Foil, Textile........................... 245
Feeding, Infant...................... 254
Faculties, College, Asso. of....... 481
Gums, Lancing of the............. 69
Gold Leaf.............................. 73
" Precipitation of............... 93
Glycerine as an Excipient........ 146
Glossitis, Rheumatic............... 196
Gastroscope.......................... 197
Hamamelis in Epistaxis of Typhoid Fever..... ............... 143
Heart Beats........................... 145
Hurry and Worry. ................. 193
Hair, About Gray................... 198
Hydrogen, Peroxide of............ 202
Hygiene, Report of Common.... 268
Hygiene ............................... 269
Hymenial ............................. 375
Histology, Anatomy and Microscopy............................... 515
International Medical Congress. 55, 260, 370....................... 545
Idunium ............................... 93
Iodine.................................. 199
Iodol....................................627
Insects and Flowers................ 200
Illinois Society................ 212, 596
Iowa Society.. ........................ 212
Infant Feeding....................... 254
Iowa University, Dent. Dept..... 420
Indiana Dental College ........... 420
Iodoform............................... 580
Japanese Teeth...................... 255
Kansas Dental Law................. 405
" City Dental College...... 421
Listerine ......... .................... 54
Legislation ........................... 156
Law, Dental........208, 403, 405, 408.............. .................. 411
Literature Dental, Report on... 315
Law, Medical of Indiana......... 474
Literature and Nomenclature... 505
Miller's Theory...................... 40
" " Review of by Author ........................... 43
Mississippi Valley Association Dental Surgeons....106, 161, 207 ............................ 213
Maxillary Sinus, Diseased........ 155
Menthol ........................ 200, 628
Maxilla, Excision of the Superior.............................. 201
Mad River Society............212, 354
Medical Congress, International 55, 260............................ 370
" Association American, Proceedings...................... 386
Minnesota Dental Law............ 408
Morphology.......................... 627

Michigan University, Dental College of........................ 417
Maryland University, Dental Department..................... 419
Minnesota College Hospital Dental Department........... 421
Missouri Dental College........... 421
Management of Children in Dental Chair.................... 443
Minnesota Dental Society, Proceedings of....................... 468
Medical Law of Indiana.......... 474
Microscopy, Anatomy and Histology ......................... 515
Membrane, Alveolar Dental..... 523
Memoriam ............................ 565
Neuralgia, Transfer of ............. 104
Nerve Canals in Teeth, Treat-
Nerve Matter........................... 600
ment and Filling of........... 109
Necrology............ ......160, 335, 565
Nerve Stretching for Neuralgia.. 256
Neuralgia, Stretching of Nerve for.................................. 256
Nutrition in Early Life as Affecting the Teeth ............... 424
Nomenclature and Literature... 505
New Metal, Another................ 472
Norwegium ............................ 528
Oxyphosphate of Zinc........147, 341
Ohio State Dental Society Reorganized.........159, 480, 528, 529, 582.......................... 611
Operative Dentistry 10, 238, 509, 604
" " Inadequacy of our Resources in......... 238
Ohio, Northern Society of......... 264
Obituary.....................335, 426, 565
Oral Deformity....................... 377
Proceedings — Northwestern Dental Association........... 13
" Ohio State Dental Society.....................14, 611
" Miss. Valley Society .........................161, 213
" Southern Dental Association...................... 265
" Mad River Dental Society...................... 354
" American Medical Association................ 386
" Minnesota Dental Society...................... 468
" Association Dental College Faculties.......... 481
Proceedings — American Dental Association............488, 533
" British Dental Association ..................... 568
" Virginia State Association ...................... 624
Physician, Ideal...................... 87
Palate, Plastic Operations on the 142
Periostitis ................................ 148
Pyorrhœa Alveolaris.........213, 548
Pennsylvania State Society...... 371
Philadelphia Dental College..... 417
Penn. University, Department of Dentistry....................... 418
Pulpless Teeth, Treatment of.... 433
Phosphates, Earthy................. 491
Pyorrhœa and Sponge-grafting.. 550
Plates .................................... 557
Quackery, a Protest Against..... 234
Rheumatic Glossitis............... 196
Rest with Effort, Alernation of.. 396
Rubber-work, New Method of Vulcanite........................ 556
Singular Case......................... 11
Salicylic Acid as a Prophylactic Against Cholera........... 47
Shortsightedness..................... 48
Salmagundi..49, 97, 150, 203, 257, 327, 365, 422, 472, 526, 578, 628
Steel Uniformity..................... 90
Selections.........40, 69, 122, 193, 249
Sponge Gratting.........122, 197, 550
Sinus, Maxillary, Diseased....... 155
Southern Dental Asso.........158, 335
Society—Illinois .................... 212
" Iowa ........................ 212
" Kentucky State......... 372
" Mad River.........212, 264
" Michigan State........... 160
" New Jersey State...... 371
" Ohio State...159, 480, 528, 529..............................582
" Pennsylvania State..... 371
" State Indiana............ 334
" State of New York..... 264
Sanitas ...........................186, 227
Sleeplessness ........................ 195
Stains on the Teeth, Removal of 492
Sickness, Responsibility for...... 256
Soothing Syrups..................... 257
Skeletons, Trade in..................332
Teeth, Diseases of, in Relation to Reflex Maladies............ 126
" and the Womb, Relations Between ........................ 194

Teeth, Civilization and the....... 83
" Stains on, Removal of... 249
" Bleaching.................... 250
" Extraction of, for Purpose of Uniformity........... 255
" Japanese .................... 255
" Pulpless, Treatment of... 433
" In the Nose................. 473
" Artificial, may do Damage 475
Trains, Running for................. 199
Toothpicks ........................... 206
Textile Foil........................... 245
Tooth Substance, Reckless Sacrifice of........................... 282
Tennessee, Dental Department, University of.................. 416
Treatment of Deepseated Abscesses............................ 453
Usefulness, How to Increase Your 306
Unusual Case......................... 372
University of Tennessee, Dental Department..................... 416
" of Michigan, Dental Department..................... 417
" of Penn., Dental Department ........................ 418
" Vanderbilt, Dental Department ..................... 418
" Maryland, Dental Department ...................... 419
" of Iowa, Dental Department ......................... 420
" of California, Dental Department ..................... 422
" National, Dental Department ......................... 422
Vanderbilt University, Dental
Virginia State Association........ 624
Department ...................... 418
Vulcanite Rubber-work, New Method of........................ 556
Wisconsin Dental Law............ 411
Zinc, Oxyphosphate of......147, 341

### DIRECTORY OF DENTAL SOCIETIES.

*American Dental Association*—Meets on the 1st Tuesday of August, 1885, at Minneapolis, Minn. Pres. J. N. Crouse, Chicago, Ill.; Cor. Sec. A. W. Harlan, Chicago; Rec. Sec. Geo. H. Cushing, Chicago.

### STATE DENTAL SOCIETIES.

*Alabama Dental Association*—Meets annually. Next meeting at Montgomery, on the second Tuesday of April, 1885. Pres. W. R. McWilliams, Athens; Sec., C. A. Merrill, Birmingham.

*California State Dental Association*—Tuesday, May 22d, 1885. Pres. Wm. Dutch, San Francisco; Sec. S. E. Goe., San Francisco; Cor. Sec. H. E. Knox, San Francisco.

*Connecticut State Dental Society*—Pres. R. W. Brown, New London; Rec. Sec. Geo. L. Parmelee, Hartford. Annual meeting third Tuesday in May, 1885, at Hartford.

*Dental Society of the State of Maryland and District of Columbia*—Meets annually. Next meeting at Washington City, on the second Tuesday in October, 1885. Pres. B. F. Coy, of Baltimore: Sec. M. W. Foster, M. D.

*Georgia State Dental Society*—Meets second Tuesday in May, 1885, at Savannah, Ga. Pres A. G. Bonton, Savannah; Rec. Sec. G. W. H. Whitaker, Sandersville; Cor. Sec. L. D. Carpenter, Atlanta.

*Illinois State Dental Society*—Meets annually. Pres. H. H. Townsend, Pontiac; Sec. J. W. Wassal, Chicago. Next place of meeting, Peoria, second Tuesday in May, 1885

*Indiana State Dental Society*—Meets next year at Indianapolis, on the last Tuesday of June, 1885. Pres. J. E. Cravens, Indianapolis; Sec. Robert Van Valzah, Terre Haute.

*Iowa State Dental Society*—Meets annually. Next meeting in Council Bluffs, on the first Tuesday in May, 1885. Pres. E. E. Hughes, Des Moines; Rec. Sec. J. B. Montfort, Fairfield.

*Kentucky State Dental Association*—Meets annually, first Tuesday in June, 1885. Next meeting in Louisville. Pres. Wm. Van Antwerp, Mt. Sterling; Sec. E. C. Dunn, Louisville.

*Kansas State Dental Association*—Pres. Dr. J. A. Young, Emporia; Sec. C. B. Reed, Topeka. Next meeting will be held at Topeka, Kansas, in joint session with the Nebraska State Society, first Tuesday in May, 1885.

*Maine Dental Society*—Meets in August, at Thomaston. Pres. E. J. Robert, Augusta, Sec. C. E. Hussey, Beddeford.

*Maryland State Dental Association*—Meets annually next November 13. Pres. T. J. Smithers; Cor. Sec. Wm. A. Mills; Rec. Sec. B. M. Hopkinson.

*Massachusetts Dental Society*—Meets annually in Boston, second Thursday in December. Pres. F. Searl, Springfield; Rec. and Cor. Sec. W. E. Page, Boston.

*Michigan State Dental Association*—Meets annually. Next meeting at Detroit, last Wednesday in March, 1885. Pres. J. Lathrop, Detroit; Sec. J. B. McGregor, Port Huron; Treas. H. K. Lathrop, Detroit.

*Mississippi State Dental Association*—Will meet third Tuesday of June, 1885, at Jackson. Pres. Geo. W. Rembert; Sec. R. G. Miller, Jackson.

*Minnesota Dental Association*—Meets annually. Meets May 26, 1885, at Winona. Pres. W. F. Lewis, Sec. C. M. Bailey, Minneapolis.

*Missouri Dental Association*—Meets annually on the second Tuesday of July, 1885, Next meeting at Sweet Springs. Pres. D. J. McMillen; Sec. Geo. L. Shepard, St. Louis.

*Nebraska State Dental Society*—Meets annually. Next meeting first Tuesday in May 1885, at Hiawatha, Kansas, in joint session with the Kansas State Dental Society. Pres. J. W. Funck, Beatrice; Sec. W. F. Roseman, Fremont.

*New Jersey State Dental Society*—Meets annually. Next meeting at Long Branch. on Wednesday, July, 20th 1885. Pres. Dr. J, W. Scarborough, of Lambertville; Sec. C. A. Meeker, Newark.

*New Hampshire Dental Society*—Pres. D. W. Eagerly, Farmington; Sec. C. W. Clerment Manchester.

*North Carolina State Dental Society*—Meets annually. Next meeting at Raleigh, second Tuesday in June, 1885. Pres. W. H. Hoffman; Sec. Thos. M. Hunter, Fayetteville.

*Ohio State Dental Society*—Meets annually. Next meeting at Chillicothe, last Wednesday of October, 1885. Pres. C. H. James, Cincinnati; Sec. J. R. Callahan, Hillsboro.

*Oregon State Dental Society*—Meets annually. Pres. J. R. Cardwell, Portland; Rec. Sec. S. J. Barber.

*Pennsylvania State Dental Society*—Meets Tuesday, July 27th, 1885, at Cresson Springs; Pres. Geo. Elliot, Meadville; Rec. Sec. E. P. Kremer, Lebanon, Pa.; Cor. Sec. W. H. Fundenberg, Pittsburgh.

*Rhode Island Dental Association*—Pres. A. W. Buckland, Woonsocket; Sec. L. L. Buckland, Providence.

*South Carolina State Dental Association*—Meets annually. Pres. B. A. Muckenfuss; Sec. G. F. S. Wright, Columbia; Cor. Sec. R. A. Smith. Next meeting at Cheraw, May 3, 1885.

*Texas State Dental Association*—Meets in the city of Galveston, May 4, 1885. Pres. Geo. S. Staples, of Sherman; Sec. G. M. Patten, Huntsville, Texas.

*Tennessee Dental Association*—Meets Annually Next meeting at Nashville third Tuesday in February, 1885. Pres. J. W. Lee, Chattanooga; Rec. Sec. D. B. Blakemore, Nashville · Cor. Sec. R. B. Lees, Nashville.

*The Dental Society of the State of New York*—Meets annually on the second Wednesday in May. Next session at Albany, May 12, 1885. Pres. L. S. Straw, Newburgh; Rec. Sec. J. Ewd. Line, Rochester; Cor. Sec. W. H. Atkinson, New York.

*Vermont State Dental Society*—Meets annually. Next meeting at Burlington, March 15, 1885. Pres. R. M. Chase, Bethel; Sec. Thos. Mound, Rutland.

*Virginia State Dental Association*—Meets in Charlottsville, Tuesday, August 19, 1885. Pres. J. Hall Moore; Sec. L. M. Cowarden, Richmond.

*Wisconsin State Dental Society*—Meets annually. Next meeting at Milwaukee, July 20, 1885 Pres. B. G. Marklein, Milwaukee; Sec. C. A. Southwell, Appleton, Wis.

### LOCAL SOCIETIES.

*American Academy of Dental Science*—Meets annually in Boston, Mass., on the last Wednesday in October, 1885. Pres. J. L. Williams; Rec. Sec. Jno. T. Codman; Cor. Sec. C. P. Wilson, Boston.

*Brooklyn Dental Society and Second District Society United*—Meets quarterly—Pres. T. H. Holly; Rec. Sec. A. N. Russell, Brooklyn.

*Central Dental Association of Northern New Jersey*—Meets annually in February. Pres. C. A. Meeker, Newark, Sec. G. Carleton Brown, Elizabeth.

*Central Illinois Dental Society*—Meets annually. Next meeting at El Paso, Oct. 9, 1885; Pres. ——; Sec. C. R. Taylor, Streetor.

*Chicago Dental Society*—Meets monthly. Pres. A. W. Harlan; Sec. Jas. G. Reid.

*Cincinnati Dental Society*—Meets on the second Tuesday in each month. Pres. Frank Bell; Sec. W. N. Williams.

*Connecticut Valley Dental Society*—Meets 27 and 28 of Oct. 1885, at Savin Rock Conn Pres. C. T. Stockwell, Springfield, Mass. Sec. A. M. Ross, Chicopee.

*Eighth District Dental Society, N. Y.*—Meets semi-annually. Next meetlng in Buffalo, April 15, 1885. Pres. S. A. Freeman; Sec. C. S. Butler, Buffalo.

*East Tennessee Dental Association*—Meets annually. Next meeting will be held at Chattanooga, Tenn., on the first Tuesday of Aug., 1885. Pres. R. A. B. Noyers, Sec. S. N. Prothro.

*New Hampshire State Dental Society*—Meets annually. Pres. H. Hill, Manchester; Sec. E. B. Davis, Concord.

*Mad River Dental Society*—Meets annually. Next meeting at Dayton, Tuesday, May 19, 1885. Pres. G. L. Paine, Xenia; Sec., W. M. Sillito, Xenia.

*Mississippi Valley Dental Society*—Meets annually at Cincinnati, on first Wednesday in March, 1886. Pres. A. F. Emminger, Columbus, O.; Sec. A. G. Rose, Cincinnati.

*New England Dental Society*—Meets annually first Thursday and Friday in October, in Boston. Pres., Wm. Barker; Sec., A. M. Dudley.

*Nashville Dental Association*—Meets on the second Tuesday of each month. Pres. R. R. Freeman; Sec. L. G. Noel, Nashville.

*Northern Ohio Dental Association*—Meets annually. Next meeting at Cleveland, O. on the second Tuesday in May, 1885. Pres. D. Gale French of Pittsburg, Pa.; Rec. Sec. Geo. H. Wilson, Painesville, Cor. Sec. H. F. Harvy, Cleveland, O.

*Northwestern Dental Association* (embraces Dakota, Minnesota and Manitoba) meets annually. Next meeting at Frago, Dakota, fourth Tuesday in July, 1885. Pres. Lewis Ottofy, Otoffy, Dak.; Sec. S. J. Hill, Fargo, Dak.

*Ohio Dental College Association*—Meets annually, Tuesday, March 2, 1885, at Cincinnati. Pres. H. M. Reid, Cincinnati; Rec. Sec. F. A. Hunter, Cincinnati.

*Odontological Society of Pennsylvania*—Meets on the first Wednesday of each month, at 8 o'clock p. m., in the Philadelphia Dental College. Pres. J. L. Eisenbrey, Rec. Sec. Ambler Tees.

*Odontological Society of New York City*—Meets the third Tuesday of each month. Pres. S. G. Perry; Rec. Sec. E. T. Payne.

*Pennsylvania Association of Dental Surgeons*—Pres. J. H. Githens. Rec. Sec. Theo. F. Chupein.

*Pittsburg Dental Society*—Meets the first Tuesday Evening of each month. Pres. F. A. Reinhart; Sec. W. N. Fundenburgh.

*Fifth District Dental Society of N. Y.*—Meets semi-annually. Next meeting in Syracuse, on the 12th of October, next. Pres. E. L. Swartwout, of Utica. Sec. I. C. Curtis of Fulton.

*San Francisco Dental Society*—Meets monthly. Pres. Alex. Warner: Sec. A. W. Knowles; Treas. J. J. Birge.

*Sixth District Dental Society of N. Y.*—Meets semi-annually. Next meeting at Cortland, Thursday, Oct. 6th, 1885. Pres. M. D. Jewett, Richford Springs; Sec. E. D. Downs, Owego.

*St. Louis Dental Society*—Meets first Tuesday in each month. Pres. J. B. Newby; Sec. J. W. Whipple.

*Washington Dental Society*—Meets second Tuesday of each month. Pres. R. Finley Hunt; Rec. Sec. H. M. Schooly.

*Southern Dental Association*—Meets in New Orleans, La., on the 31st of March, 1885. Pres. A. O. Rawls; Cor. Sec. J. P. Holmes, Macon, Ga.; Sec. W. H. Hoffman.

*First District Dental Society of the State of New York*—Meets annually. Pres. A. L. Northrup; Sec. J. E. Dexter, New York.

### EXCHANGES.

St. Louis, Medical and Surgical Journal.
The Pacific Medical and Surgical Journal, San Francisco.
Cincinnati Lancet and Clinic.
Dental Cosmos, Philadelphia.
Chicago Medical Examiner.
The Detroit Review of Medicine and Pharmacy.
Medical Record, N. Y.
American Journal of Dental Science, Baltimore.
Indiana Journal of Medicine.
Archives of Dentistry.
The Sanitaran, New York City.
L'art Dentaire, Paris.
Le Progress Dentare, Paris.
Deutische Monatsschrift fuer Zahnheilkunde Leipzig.—Arthur Felix.
Physician and Surgeon, Ann Arbor, Mich.
Ohio State Journal of Dental Science, Xenia, Ohio
The Microscope, Ann Arbor, Mich.
Scientific American, New York.
Independent Practitioner, N. Y.
The Dental Record, London.
The Journal of the British Dental Association, London.
The Southern Dental Journal.
Items of Interest, Philadelphia.
The Dental Practitioner.—The Cincinnati "Medical" News.
The New England Journal of Dentistry.
The British Journal of Dental Science.

### Cincinnati College of Medicine and Surgery,

**164 GEORGE ST., CINCINNATI, O.**

### FACULTY.

R. C. STOCKTON REED, M.D., DEAN, *Professor of Materia Medica, Therapeutics, and State Medicine.*
GEORGE B. ORR, M.D., *Professor of Principles and Practice of Surgery and Clinical Surgery.*
CHAS. A. LEE REED, M.D., *Professor of Obstetrics and Gynæcology.*
MARION L. AMICK, M.D., *Professor of Anatomy.*
J. H. HAZARD, M.D., SECRETARY, *Professor of Physiology.*
J. B. HAIGHT, M.D., *Professor of Principles and Practice of Medicine.*
JOHN BOHLANDER, M.D., *Professor of Chemistry and Toxicology.*
W. R. AMICK, M.D., *Professor of Ophthamology and Otology.*
L. C. CARR, M.D., *Professor of Diseases of Children and Midwifery.*
ANDERSON N. ELLIS, M.D., *Professor of Laryngology.*
W. K. BOYLAN, M.D., *Professor of Materia Medica and Therapeutics.*
JOSEPH WATSON, M. D., *Professor of Anatomy and Histology.*
FRANK W. SAGE, D.D.S., *Professor of Oral Surgery.*
WM. F. TAYLOR, M.D , *Professor of Dermatology, Genito-Urinary and Veneral Diseases.*
LOUIS W. SAUER, PH. G., *Professor of Pharmacy.*
JOHN. G. REED, M.D., *Demonstrator of Anatomy.*

### THE DENTAL COLLEGE

OF THE

### University of Michigan.

The Eleventh Annual Session of this Institution will commence on the 1st of October and close on the last Thursday of June, thus making a course of nine months. The regular course of instruction commences at once, and will continue through the term, with the customary vacation.

The students in this department will receive instruction in Anatomy, Physiology, Pathology, Chemistry, Materia Medica, Therapeutics, and Surgery, from the Professors of their respective branches in the *Department of Medicine and Surgery* of the University, where lectures commence, and continue the same as with the Dental College. There will also, in addition, be a special course upon Oral Pathology and Surgery, which will embrace from thirty to thirty-five lectures.

### FACULTY OF THE DENTAL DEPARTMENT.

| | |
|---|---|
| J. B. ANGELL, LL.D. | President. |
| J. TAFT, M.D., D.D.S. | Principles and Practice of Operative Dentistry. |
| CORYDON L. FORD, M.D., D.D.S. | Anatomy and Physiology. |
| J. A. WATLING, D.D.S. | Clinical and Mechanical Dentistry. |
| W. H. DORRANCE, DD.S. | Prosthetic Dentistry. |
| J. N. Martin, M.D., | Lecturer on Oral Pathology and Surgery. |
| MARGARET HUMPHREYS, D.D.S. | Demonstrator of Clinical Dentistry. |
| ———————— | Demonstrator of Prosthetic Dentistry. |

Students should be promptly present at the College on Wednesday, September 30th, at 10 o'clock, A.M., to make the preliminary arrangements for entering upon regular work. Seats in the lecture room are assigned by election to students in the order of registration on the Steward's Books; and each student is expected to occupy during the session such seat as he may select. Students, on arriving in Ann Arbor, should call at the Steward's office.

### CONDITIONS OF GRADUATION.

The candidate must be twenty-one years of age. *He must furnish satisfactory evidence of good moral character.*

He must devote three years to the study of his profession. He must attend two full courses of lectures in the Dental College, or one in some college having an equal standard of requirements, and the last one here, and we recommend that he attend three courses regularly.

He must sustain an examination satisfactory to the Faculty in all the branches taught.

A graduate of the Medical College may enter the Senior Class, and, if found qualified, may graduate after two years have been devoted to the study of dentistry.

### FEES AND EXPENSES.

The fees, which must be paid in advance, are as follows:

RESIDENTS OF MICHIGAN.—Matriculation fee, $10.00; annual dues, $25.00

NON-RESIDENTS.—Matriculation, $25.00; annual dues, $35.00.

GRADUATION FEE.—For all alike, $10.00. The admission fee is paid but once, and entitles the student to the privileges of permanent membership in any department of the University. The annual dues are paid the first year and every year thereafter while at the University.

For further particulars, address the Dean of the Dental College, Ann Arbor, Michigan.

**J. TAFT, Dean.**

July-85-6m.

### THE DENTAL COLLEGE

OF THE

### University of Michigan.

The Eleventh Annual Session of this Institution will commence on the 1st of October and close on the last Thursday of June, thus making a course of nine months. The regular course of instruction commences at once, and will continue through the term, with the customary vacation.

The students in this department will receive instruction in Anatomy, Physiology, Pathology, Chemistry, Materia Medica, Therapeutics, and Surgery, from the Professors of their respective branches in the *Department of Medicine and Surgery* of the University, where lectures commence, and continue the same as with the Dental College. There will also, in addition, be a special course upon Oral Pathology and Surgery, which will embrace from thirty to thirty-five lectures.

### FACULTY OF THE DENTAL DEPARTMENT.

J. B. ANGELL, LL.D. - - - - - - - - - - - President.
J. TAFT, M.D., D.D.S. Principles and Practice of Operative Dentistry.
CORYDON L. FORD, M.D., D.D.S. - - - Anatomy and Physiology.
J. A. WATLING, D.D.S. - - - Clinical and Mechanical Dentistry.
W. H. DORRANCE, DD.S. - - - - - - - Prosthetic Dentistry.
J. N. Martin, M.D., - - - Lecturer on Oral Pathology and Surgery.
MARGARET HUMPHREYS, D.D.S. - Demonstrator of Clinical Dentistry.
———————————— Demonstrator of Prosthetic Dentistry.

Students should be promptly present at the College on Wednesday, September 30th, at 10 o'clock, A.M., to make the preliminary arrangements for entering upon regular work. Seats in the lecture room are assigned by election to students in the order of registration on the Steward's Books; and each student is expected to occupy during the session such seat as he may select. Students, on arriving in Ann Arbor, should call at the Steward's office.

### CONDITIONS OF GRADUATION.

The candidate must be twenty-one years of age. *He must furnish satisfactory evidence of good moral character.*

He must devote three years to the study of his profession. He must attend two full courses of lectures in the Dental College, or one in some college having an equal standard of requirements, and the last one here, and we recommend that he attend three courses regularly.

He must sustain an examination satisfactory to the Faculty in all the branches taught.

A graduate of the Medical College may enter the Senior Class, and, if found qualified, may graduate after two years have been devoted to the study of dentistry.

### FEES AND EXPENSES.

The fees, which must be paid in advance, are as follows:

Residents of Michigan.—Matriculation fee, $10.00; annual dues, $25.00

Non-Residents.—Matriculation, $25.00; annual dues, $35.00.

Graduation Fee.—For all alike, $10.00. The admission fee is paid but once, and entitles the student to the privileges of permanent membership in any department of the University. The annual dues are paid the first year and every year thereafter while at the University.

For further particulars, address the Dean of the Dental College, Ann Arbor, Michigan.

J. TAFT, Dean.

July-85-6m.

### THE DENTAL COLLEGE

OF THE

### University of Michigan.

The Eleventh Annual Session of this Institution will commence on the 1st of October and close on the last Thursday of June, thus making a course of nine months. The regular course of instruction commences at once, and will continue through the term, with the customary vacation.

The students in this department will receive instruction in Anatomy, Physiology, Pathology, Chemistry, Materia Medica, Therapeutics, and Surgery, from the Professors of their respective branches in the *Department of Medicine and Surgery* of the University, where lectures commence, and continue the same as with the Dental College. There will also, in addition, be a special course upon Oral Pathology and Surgery, which will embrace from thirty to thirty-five lectures.

### FACULTY OF THE DENTAL DEPARTMENT.

J. B. ANGELL, LL.D. - - - - - - - - - - - - President.
J. TAFT, M.D., D.D.S. Principles and Practice of Operative Dentistry.
CORYDON L. FORD, M.D., D.D.S. - - - Anatomy and Physiology.
J. A. WATLING, D.D.S. - - - Clinical and Mechanical Dentistry.
W. H. DORRANCE, DD.S. - - - - - - - Prosthetic Dentistry.
J. N. Martin, M.D., - - - Lecturer on Oral Pathology and Surgery.
MARGARET HUMPHREYS, D.D.S. - Demonstrator of Clinical Dentistry.
———————— Demonstrator of Prosthetic Dentistry.

Students should be promptly present at the College on Wednesday, September 30th, at 10 o'clock, A.M., to make the preliminary arrangements for entering upon regular work. Seats in the lecture room are assigned by election to students in the order of registration on the Steward's Books; and each student is expected to occupy during the session such seat as he may select. Students, on arriving in Ann Arbor, should call at the Steward's office.

### CONDITIONS OF GRADUATION.

The candidate must be twenty-one years of age. *He must furnish satisfactory evidence of good moral character.*

He must devote three years to the study of his profession. He must attend two full courses of lectures in the Dental College, or one in some college having an equal standard of requirements, and the last one here, and we recommend that he attend three courses regularly.

He must sustain an examination satisfactory to the Faculty in all the branches taught.

A graduate of the Medical College may enter the Senior Class, and, if found qualified, may graduate after two years have been devoted to the study of dentistry.

### FEES AND EXPENSES.

The fees, which must be paid in advance, are as follows:

RESIDENTS OF MICHIGAN.—Matriculation fee, $10.00; annual dues, $25.00

NON-RESIDENTS.—Matriculation, $25.00; annual dues, $35.00.

GRADUATION FEE.—For all alike, $10.00. The admission fee is paid but once, and entitles the student to the privileges of permanent membership in any department of the University. The annual dues are paid the first year and every year thereafter while at the University.

For further particulars, address the Dean of the Dental College, Ann Arbor, Michigan.

**J. TAFT, Dean.**

July-85-6m.

### THE DENTAL COLLEGE

OF THE

### University of Michigan.

The Eleventh Annual Session of this Institution will commence on the 1st of October and close on the last Thursday of June, thus making a course of nine months. The regular course of instruction commences at once, and will continue through the term, with the customary vacation.

The students in this department will receive instruction in Anatomy, Physiology, Pathology, Chemistry, Materia Medica, Therapeutics, and Surgery, from the Professors of their respective branches in the *Department of Medicine and Surgery* of the University, where lectures commence, and continue the same as with the Dental College. There will also, in addition, be a special course upon Oral Pathology and Surgery, which will embrace from thirty to thirty-five lectures.

### FACULTY OF THE DENTAL DEPARTMENT.

| | |
|---|---|
| J. B. ANGELL, LL.D. - - - - - - - - - - - - - | President. |
| J. TAFT, M.D., D.D.S. | Principles and Practice of Operative Dentistry. |
| CORYDON L. FORD, M.D., D.D.S. - - - | Anatomy and Physiology. |
| J. A. WATLING, D.D.S. - - - | Clinical and Mechanical Dentistry. |
| W. H. DORRANCE, DD.S. - - - - - - - | Prosthetic Dentistry. |
| J. N. Martin, M.D., - - - | Lecturer on Oral Pathology and Surgery. |
| MARGARET HUMPHREYS, D.D.S. - | Demonstrator of Clinical Dentistry. |
| ———————— | Demonstrator of Prosthetic Dentistry. |

Students should be promptly present at the College on Wednesday, September 30th, at 10 o'clock, A.M., to make the preliminary arrangements for entering upon regular work. Seats in the lecture room are assigned by election to students in the order of registration on the Steward's Books; and each student is expected to occupy during the session such seat as he may select. Students, on arriving in Ann Arbor, should call at the Steward's office.

### CONDITIONS OF GRADUATION.

The candidate must be twenty-one years of age. *He must furnish satisfactory evidence of good moral character.*

He must devote three years to the study of his profession. He must attend two full courses of lectures in the Dental College, or one in some college having an equal standard of requirements, and the last one here, and we recommend that he attend three courses regularly.

He must sustain an examination satisfactory to the Faculty in all the branches taught.

A graduate of the Medical College may enter the Senior Class, and, if found qualified, may graduate after two years have been devoted to the study of dentistry.

### FEES AND EXPENSES.

The fees, which must be paid in advance, are as follows:

Residents of Michigan.—Matriculation fee, $10.00; annual dues, $25.00

Non Residents.—Matriculation, $25.00; annual dues, $35.00.

Graduation Fee.—For all alike, $10.00. The admission fee is paid but once, and entitles the student to the privileges of permanent membership in any department of the University. The annual dues are paid the first year and every year thereafter while at the University.

For further particulars, address the Dean of the Dental College, Ann Arbor, Michigan.

**J. TAFT, Dean.**

July-85-6m.

### CONTENTS.

COMMUNICATIONS.

Pathology of Alveolar Abscess.......... 585

Alveolar Abscess and Treatment of Dead Teeth.......... 590

Hydrochlorate of Cocaine.......... 593

PROCEEDINGS.

Illinois State Dental Society.......... 596

Ohio State Dental Society.......... 611

Virginia State Dental Association.......... 624

Report of the Ohio State Dental Examining Board.......... 626

SELECTIONS.

Iodol.......... 627

Morphology.......... 627

SALMAGUNDI.......... 628

EDITORIAL.

Ohio State Dental Society.......... 632

International Medical Congress.......... 633

Bibliographical.......... 636

---

### THE DENTAL REGISTER,

A MONTHLY MAGAZINE DEVOTED TO THE INTERESTS OF THE

### DENTAL PROFESSION.

*Terms Reduced to $2.00 Per Year. Single Copies, 25 Cents.*

### EDITED BY PROF. J. TAFT, D.D.S.

—AND—

### Published by SAM'L A. CROCKER & CO., Ohio Dental and Surgical Depot.

The volume commences in January and closes in December.

The REGISTER being issued monthly is always fresh, therefore Indispensable to the practitioner who desires to keep posted up with the new inventions and improvements which are daily developed. Neither expense nor effort will be spared to give value and variety to its contents. Articles will be illustrated with well executed engravings whenever they will add to the interest or assist to explanation.

Its extensive circulation and frequency of issue make it one of the BEST DENTAL ADVERTISING MEDIUMS in the United States.

All subscriptions must begin with January or July.

Local or special notices, five lines or less, will be inserted for $3 each insertion; over five lines, 50 cts. per line.

All advertising bills payable in advance, or at furthest, after the issue of the first number containing the advertisement. All transient advertisements to be paid for in advance.

☞CONTRIBUTIONS SOLICITED.

Exclusively Editorial Communications should be addressed to the Editor.

The latest number of the REGISTER may be ordered with or without other goods at any time, and all letters should be addressed to

SAM'L A. CROCKER & CO., Ohio Dental and Surgical Depot, Cincinnati, O.

